# Blue Rubber Bleb Nevus Syndrome With Multiple Cavernoma-Like Lesions on MRI: A Familial Case Report and Literature Review

**DOI:** 10.3389/fneur.2020.00176

**Published:** 2020-04-07

**Authors:** García Anwár, Paredes-Aragón Elma, Jorge-de Saráchaga Adib, Meyer-Nava Ilse, Gutiérrez-Romero Alonso, Salinas Lara Ciltlaltepelt, Novelo Soto Alma, Vega Memije Maria Elisa, Arauz Antonio

**Affiliations:** ^1^Department of Neurology, Instituto Nacional de Neurología y Neurocirugía “Dr. Manuel Velasco Suarez”, Mexico City, Mexico; ^2^Dermatology Department, Instituto Dermatológico de Jalisco “José Barba Rubio”, Guadalajara, Mexico; ^3^Departament of Neuropathology, Instituto Nacional de Neurología y Neurocirugía “Dr. Manuel Velasco Suarez”, Mexico City, Mexico; ^4^Dermatology Department, General Hospital “Dr. Manuel Gea Gonzalez”, Mexico City, Mexico

**Keywords:** Bean's syndrome, blue rubber bleb nevus syndrome, blue rubber bleb angiomatosis, blue rubber-bleb nevus, central nervous system venous malformations, central nervous system bleeding, cavernomas, BRBNS

## Abstract

Blue rubber bleb nevus syndrome (BRBNS), also called Bean's syndrome, is a rare disease associated with multiple venous malformations in the skin and gastrointestinal (GI) tract. Dermatological lesions, which are the first clinically visible manifestations, appear as skin-colored compressible protuberances or as dark-blue venous nodules, rubbery in consistency. Central nervous system (CNS) manifestations are rare, variable, non-specific, and tend to occur late in the disease, mainly reported as seizures and focal neurological deficits secondary to compression. Most cases occur sporadically, however, an autosomal dominant inheritance pattern has been reported. A 74-year-old male with history of focal epilepsy secondary to possible neurocysticercosis presented at the emergency department due to sudden onset of aphasia, left central facial paralysis, and dysphagia secondary to catastrophic intracerebral hemorrhage. Cerebral MRI showed multiple cerebral cavernous malformations (CCM)-like lesions and, on the general exploration, multiple dark-blue nodules, rubbery in consistency. One week later he died due to complicated pneumonia; a brain autopsy was performed showing multiple vascular malformations. His son had a history of focal epilepsy presumed to be related to neurocysticercosis. He had the same skin lesions and brain MRI pattern. Histological analysis of the skin lesions of the two cases showed venous vascular malformations. A non-systematic review was carried out, in which all case reports of blue nevus syndrome with neurological manifestations in adults were included.

## Background

Blue rubber bleb nevus syndrome (BRBNS), or Bean's syndrome, is a rare vascular anomaly that is comprised of multifocal venous malformations, predominantly in the skin. The appearance of vascular malformations can be localized or diffuse and may occur in any part of the body, including the liver, lungs, spleen, muscles, eyes, genitourinary tract, and the CNS (1). In 1860, Gascoyen et al. reported the first case of multiple cutaneous and GI cavernous hemangiomas. However, the term “blue rubber nevus syndrome” was designated in 1958 by Bean, due to the distinct tactile sensation produced by the rubber lesions (1, 2).

BRBNS is characterized by the presence of vascular malformations which consist of localized developmental defects that affect arteries, veins, capillary beds, and lymphatic vessels. They can be classified into two main categories: vascular tumors and vascular malformations. Vascular malformations represent focal increments in the number of vessels, which are tortuous, lack spontaneous regression, and can remain stable with no changes in size or shape. They can be further classified as high or low flow lesions. Spontaneous bleeding is rare, although it may be caused by trauma (3, 4). Cutaneous angiomas are found in up to 93% of patients with Bean's syndrome, with GI hemangiomas in 76%, CNS lesions in 13%, liver lesions in 11%, and diverse muscles in 11% (5).

## Case 1

A 74-year-old male was admitted to the emergency department due to sudden aphasia, left facial palsy, and difficulty swallowing. Both his sister and son were diagnosed with epilepsy, presumably due to neurocysticercosis.

He was diagnosed with structural epilepsy suspected to be secondary to intraparenchymal neurocysticercosis and received a single course of albendazole. The patient had been on carbamazepine 200 mg bid for most of his life, with regular seizure control reporting a frequency of one seizure a year.

**Figure 1:**
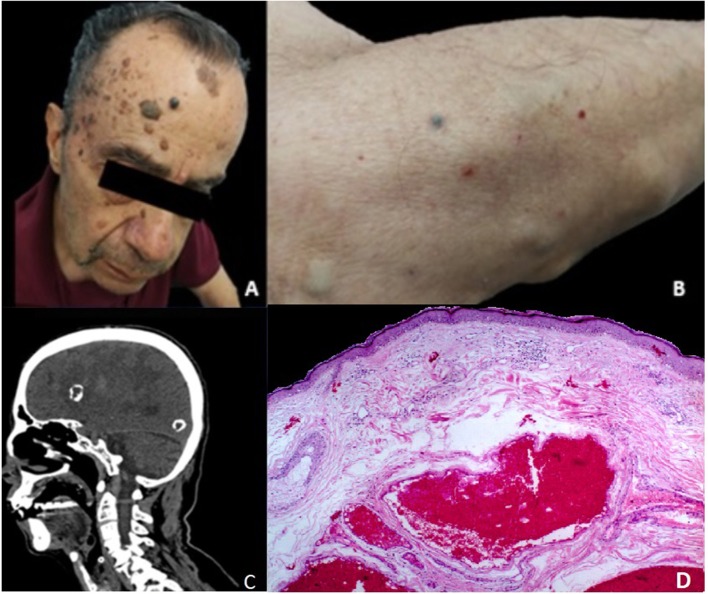
**(A)** Macroscopic view of medium and dark-blue venous nodules affecting the patient's forehead. **(B)** Skin-colored compressible protuberances and a small, dark-blue venous nodule affecting the right forearm. **(C)** A sagittal view of a cranio-cervical computed tomography scan shows heavy, hyperdense, calcified lesions centered by a hypodensity. The diffuse white matter hypodensity represents severe edema with mass effect. **(D)** Hematoxylin and eosin staining of the lesions show light areas of dilated venous vessels and congestive, thin-walled veins internally coated by a layer of flat endothelial cells. The vascular channels are arranged in the superficial, middle and deep reticular dermis. Written informed consent was obtained from the participants for the publication of this case report.

Upon examination, he had disseminated skin lesions located in the upper thorax, abdomen, and all limbs and mucous membranes. The lesions consisted of violet and skin-colored nodules and macules, ranging from 0.5 mm to 2 cm. Nodular neoformations were rubbery, adherent, and seemed to be vascular in nature; skin biopsies were taken (Figures 1A,B).

On neurological examination, the patient had bilateral abducens nerve palsy, left central facial palsy, and left soft-palate palsy, accompanied by a diminished ipsilateral gag reflex. His tongue deviated to the left upon protrusion and he had a discrete left-body weakness with ipsilateral extensor plantar response. He exhibited left-side dysmetria on finger-to-nose test.

**Figure 2:**
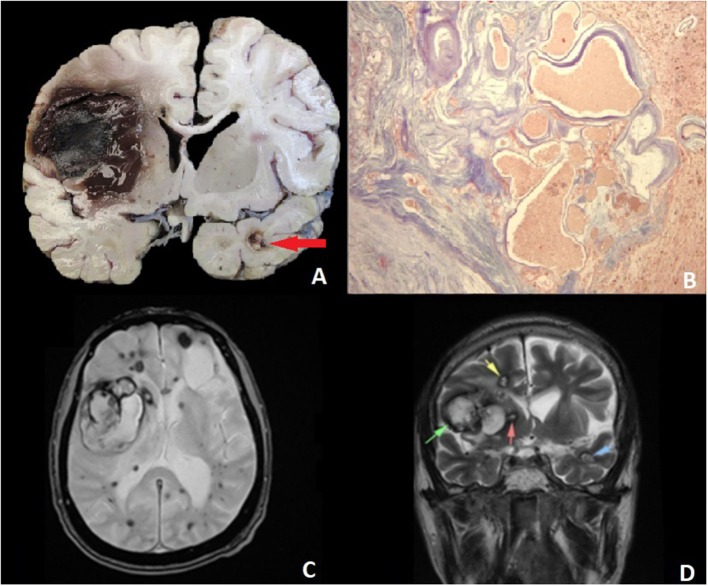
**(A)** Sagittal section of the encephalon shows a cortico-subcortical intraparenchymal hemorrhage that destroyed the basal ganglia. A nodular lesion in the temporal cortex corresponds to an AVM (arrow). **(B)** Malformed and irregular intracortical vessels. Masson's trichrome stain x. **(C)** Axial T2-weighted MR (magnetic resonance) and gradient echo (GRE) images: bilateral supratentorial hypointense lesions, harboring a central hyperintensity, 1.2 to 1.6 cm. A heterogeneous tumoral lesion can be seen in the right frontal lobe (5.38 × 5.4 cm), with mass effect and medial displacement of both basal ganglia and ipsilateral ventricle. **(D)** A coronal view of a T1-weighted image with contrast shows hyperintense lesions, primarily located in the right frontal lobe, and a similar lesion in the temporal pole corresponding to AVM (arrows).

A brain computed tomography (CT) scan revealed a large, right frontal hematoma in acute and subacute phases with perilesional edema and no deviation of the mid-line structures, multiple calcified intraparenchymal lesions, as well as a punctiform hemorrhage within the medulla oblongata (Figure 1C). A computed tomography angiography (AngioCT) of the head was performed, confirming multiple heterogeneous contrast-enhancing vascular malformations in the cortical and subcortical areas of both cerebral hemispheres. Brain MRI showed in FLAIR a moderately differentiated hyperintense lesion located in the right frontotemporal region, with irregular borders, measuring 26.7 × 37.7 mm in its largest dimension, with abundant perilesional edema and an irregular core, as well as two lesions located in the right occipital lobe measuring almost 10 mm in diameter each, and another hypointense lesion measuring 10.5 mm in its largest diameter in the left temporal pole. On T2^*^ gradient-echo (GRE), numerous supra and infratentorial punctate hypointense foci were observed. Infratentorial lesions included multiple hypointense lesions in the cerebellum, both in the vermis, and each hemisphere, a hypointense pontine lesion with a diameter of 10.5 mm, and a lesion in the midbrain that measured 6 × 5 mm. This pattern was consistent with CCM—like lesions (Figures 2C,D).

The following week, the patient presented with respiratory distress. A thorax CT was performed, which showed multilobar pneumonia. Intravenous, wide-spectrum antibiotics were initiated, and *Staphylococcus aureus* was isolated from the blood and sputum samples. He died in the next few days, and an autopsy was performed.

Histopathology of the brain tissue revealed multiple arteriovenous malformations characterized by poorly formed vessels with incomplete walls and a deposit of hyaline material, surrounded by abundant collagen and with areas of cerebral parenchyma, macrophages with hemosiderin, and vascular ectasia. These malformations had different sizes and locations; some were calcified. Additionally, there was intraparenchymal hemorrhage at the basal ganglia (Figures 2A,B).

Dermatologic histopathology reported skin lesions with multiple vascular proliferations with areas of dilated and congestive venous vessels, ectasia, and extensively hyalinized, focal, or complete disassembly of the middle tunic. A separation into inner or outer layers by an aberrant elastic lamina was also observed (Figure 1D). Based on these findings, the diagnosis of blue nevus syndrome was made.

Because this syndrome may present as an autosomal dominant inheritance, an extensive examination of the patient's relatives was performed.

## Case 2

The son of the previous patient (Case 1) was a 45-year-old male with a past medical history of epilepsy, diagnosed with CNS neurocysticercosis. He had been treated with carbamazepine 400 mg tid for several years.

**Figure 3:**
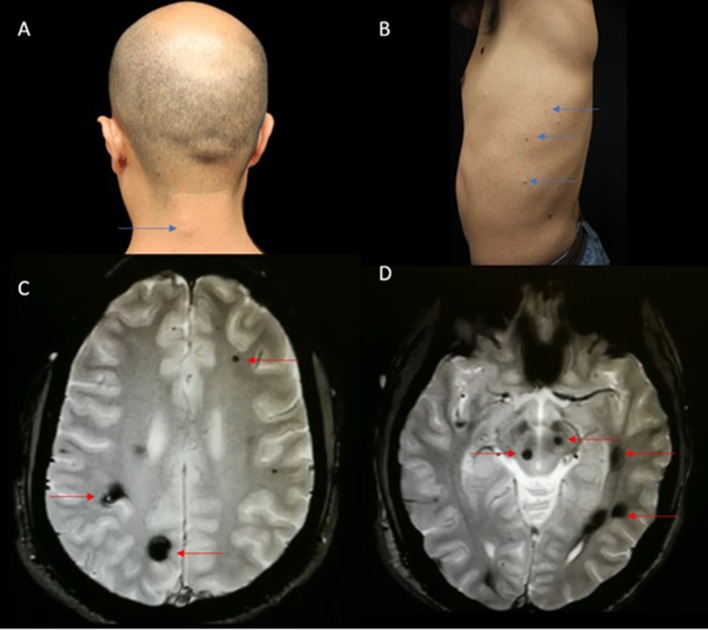
**(A)**. Macroscopic view of skin-colored compressible protuberances in the cervical neck region simulating a lipoma (Blue arrow). **(B)** Macroscopic view of the left side of the chest showing small, dark-blue venous nodules (Blue arrow). **(C,D)** Axial gradient echo images, multiple lesions shown by red arrows.

On physical examination, he presented with multiple skin lesions and was sent to dermatology, where biopsies were collected. Hematoxylin and eosin stains as well as Masson's trichromic staining showed the exact histopathological findings as in the previous case; with ectatic, congested, and thin vessels whose walls had decreased smooth muscle and abundant elastic collagen fibers. A gastroenterology assessment was requested, and a colonoscopy was performed without any meaningful pathological findings. Neurological examination was normal. Brain MRI T2-weighted GRE revealed multiple hypointense lesions, suggestive of CCM-like, with no associated acute complications (Figure 3).

Written informed consent was obtained from the participants for the publication of this case report.

The present cases provide examples of the diverse clinical presentations of this rare syndrome and highlight the clinical relevance of a complete physical examination when assessing multiple cerebral vascular malformations, as well as an intracerebral hemorrhage of apparently unknown origin. Although this syndrome is quite rare, it is an important differential diagnosis to keep in mind when approaching complex vascular scenarios.

## Discussion

The cases presented above depict relatively uncommon clinical features of BRBNS. One of them presented with epilepsy and cerebral hemorrhage, and the second one only with epilepsy. The clinical heterogeneity of this series is consistent with what has been published before. Gastrointestinal bleeding remains the most common presentation in a published series. In addition, this syndrome is typically encountered sporadically as a new mutation (2). In our cases, the genetic suspicion might involve an autosomal dominant inheritance pattern, most commonly seen in chromosome 9 (6, 7). In addition, in 2017, new somatic double (cis) mutations were discovered in the TEK gene encoding for TIE2, also known as an angiopoietin-1 receptor expressed in the endothelial cells, which is critical for endothelial-cell to smooth-muscle-cell communication in venous morphogenesis (3, 8). These variations in patterns of inheritance may influence the clinical heterogeneity of our cases. Unfortunately, no genetic assessment was performed due to the lack of this specific testing in our hospital.

The pattern found in the cerebral MRI of the two cases resembled CCM at the beginning, however, the histopathological analysis of the brain lesions revealed non-cavernomatous vascular malformations. In addition, one of these lesions grew into a considerable size and suffered spontaneous bleeding, which led to acute neurological symptoms.

## Clinical Presentation

Most patients with BRBNS are diagnosed at birth or during early infancy: 30% at birth, 9% at preschool age, 48% at infancy, 9% in adolescence, and 4% in adulthood (5). Diagnosis is almost always suspected based on characteristic cutaneous lesions. These lesions normally have a bluish-purple color, are relatively small (1 mm to 10 cm wide), are well-circumscribed, and have multiple locations. They have a rubber-like quality upon palpation. These lesions may grow throughout life, and most of them remain asymptomatic. They seem to occur frequently on the palms, soles, trunk, and perineum.

Cutaneous lesions are usually asymptomatic; however, 5% of patients report experiencing pain and 2% experience sweating.

The most common GI manifestations involve bleeding and ferropenic anemia, followed by abdominal pain due to intussusception, intestinal torsion, and rupture. Malformations can present throughout the entire GI tract, however most of them appear in the small bowel and distal colon (5, 6).

A non-systematic review was carried out, wherein these key words were used in PUBMED: ¨*blue bleb nevus syndrome*,¨ ¨*nevus syndrome*,¨ ¨*bean syndrome*,¨ *and* ¨*familial adult blue bleb nevus syndrome*.¨ All cases of blue nevus syndrome with neurological manifestations were included.

There are ~200 reports of BRBNS cases in the literature. We identified 21 case reports describing 21 patients (12 males and 9 females; total mean age: 40.2 years, male mean age 35 years, female mean age 41 years, range: 1–82 years). Clinical and brain MRI features, as well as vascular malformation findings are summarized in Table 1. They were classified into several groups according to specific neurological involvement: Chiari malformation, vascular tumors, diffuse neurological symptoms, and exclusively infratentorial neurological symptoms.

**Table 1:** Summary of Blue Rubber Bleb Nevus Syndrome Associated with Central Nervous System (CNS) involvement.

**Clinical neurological features**	**Imaging findings**	**Systemic features**	**Gender/age**	**References**
• Seizures • Chronic Headache • Vertigo • Motor weakness • Neck pain • Cognitive impairment • Cerebellar ataxia • Tinnitus • Cortical syndromes	**Brain/spinal developmental vascular anomalies** • Venous thrombosis • Non-cavernomatous venous malformations (telangiectasias, dural arteriovenous fistula, hemangiomas, angiomas) • Multiple brain cavernomas • Saccular aneurysms	• Multiple blackish-to-bluish rubbery cutaneous lesions • Skeletal anomalies • GI vascular malformations • Intussusception • Anemia • Gastrointestinal hemorrhage	F/ Mean age 41y 8 M/ Mean age 35y	(9) (10) (11) (12) (13) (14) (15) (16) (17) (18) (19) (4) (20) (21) (22) (23)
• Tinnitus • Chronic headache • Reduced hearing	• Type I Chiari malformation with tonsillar descent to the mid-C2 vertebra • Syringomyelia • Abnormal venous drainage	• Ventral septal defect at birth • Multiple blackish-to-bluish rubbery cutaneous lesions	F/14y M/26y	(24) (25)
• Orbital proptosis • Diplopia • Cranial nerve palsies	**Brain developmental vascular anomalies** • Non-cavernomatous venous malformations	• Multiple blackish-to-bluish rubbery cutaneous lesions • GI vascular malformations	M/75Y	(26)
**Unspecified tumors/Neoplasia**	**Brain/spinal developmental vascular anomalies** • Extradural masses at T2-T3, T5-T6 • Macrocystic encephalomalacia	• Multiple blackish-to-bluish rubbery cutaneous lesions • Bladder and bowel abnormalities • Sensitive symptoms • Backpain • Weakness • Anemia • Gastrointestinal hemorrhage	M/70y M/13y	(27) (28)

The main clinical presentation includes vascular lesions in the skin and gastrointestinal bleeding; CNS symptoms are infrequent (13%) (5) and have a diverse presentation. Focal neurological symptoms, subarachnoid hemorrhage, and seizures are among the features that may be present (17).

In our review, we found headaches in 42.8%, motor weakness in 23.8%, brainstem-related symptoms in 23% (most cranial nerve disturbances), seizures in 21%, visual symptoms in 9 % (hemianopia, visual distortions, and phosphenes), sensory disturbances in 31.9% (hypoesthesia or paresthesia), ataxia in 14.4%, and dizziness and hearing disturbances in 9 % of the patients.

All reported patients presented with classic skin lesions and 33.3% presented with digestive hemorrhage. Cutaneous thrombosis can develop due to low vascular flow. This same pattern can also appear in CNS lesions. Either an AngioCT or AngioMRI is often recommended for a complete assessment. Complementary tools such as electroencephalograms are also advised, as focal spike waves are often detected (29).

In our review, these MRI lesions were observed when neurological symptoms manifested: non cavernomatous vascular malformations (47.6%), CCM (23.3%), disruptions in vascular drainage (37%), hemorrhagic stroke (9.5 %), and unspecified vascular tumors (9.5 %).

CT scans reveal small, well-defined densities that can be contrast or non-contrast enhancing lesions, from no mass effect to significant perilesional edema. Hypointensities on T1 and hyperintensities on T2-weighted MRI signals have been described (18). The lesions tend to calcify, although this does not imply disease stabilization, as they retain epileptogenic potential (1). Neuroimaging studies have proven useful in diagnosing asymptomatic relatives (30).

## Histopathology

Microscopic views of the lesions show irregular and dilated veins and capillaries lined by thin endothelial cells. Thrombosis is a frequent finding. No immunohistochemical marker has yet been discovered (30).

Bean described three types of cutaneous lesions according to their clinical appearance: type I: major, obscure, red-to-purple venous malformations of 10 cm or more that may be disfiguring or obstruct vital organ systems (large cavernous lesions) (30); type II: typified by “rubber bluish nipple” lesions, characterized by nodular and rubber lesions, easily compressed by touch, followed by slow reconstitution. They are usually asymptomatic (3); and type III: blue to black punctiform macules and papules, which can be adjacent to, or over, a blue macule. They do not disappear with light pressure (30–32).

## Management and Treatment Prognosis

A high index of suspicion is required to make the diagnosis, and an initial evaluation should include laboratory tests such as complete blood count (CBC), coagulation tests, stool guaiac, and a urinary sample for the detection of occult bleeding. A further evaluation of the bleeding site is recommended, depending on the location (e.g., endoscopy, colonoscopy) (6). Endoscopy can reveal blue nodules in the intestinal mucosa, which are more prone to bleeding when compared to intestinal nodules (5).

Treatment options have been described in scarce reports. Sirolimus monotherapy was found to be effective in a pediatric GI bleeding case (2, 33). Treatment for cutaneous lesions is indicated when cosmetically unacceptable or when function is compromised. For GI manifestations, medical treatment with oral iron supplementation, steroids, interferon alpha-2, and octreotide have been shown to reduce bleeding frequency and intensity. In life-threatening cases, surgical excision as well as intestinal resection might be warranted (6). Focal seizures often require antiepileptic drug treatment. Intracranial hypertension and hemorrhage may also require surgical treatment.

Prognosis depends on the affected organs and their extent. Additional data on the syndrome and its progression are required, considering the substantial clinical heterogeneity it exhibits. Periodic neurologic evaluations and intensive monitoring are essential (1, 6).

## Differential Diagnosis

An important differential diagnosis can be made with multiple CCM, especially if they present with intracranial bleeding, seizures, or headache. CCM lesions correspond to enlarged capillary channels with a single layer of endothelium without normal mature vessel wall elements or intervening brain parenchyma (34, 35).

Unlike BRBNS, dermatological manifestations are rare, presenting in around 9% of cases; whenever present, they consist of small papules (2–3 mm). They are histologically classified as capillary malformations, hyperkeratotic cutaneous capillary venous malformation, and venous malformations. These lesions have been reported in patients with mutations in CCM1/KRIT1 (7q21-22), CCM2/malcaverina (7p13), and CCM3 / PDCD10 (3q26.1). Penetration varies: 88% in CCM1, 100% in CCM2, and 63% in CCM3. (36, 37).

On the other hand, a relationship between BRBNS and CCM has been suggested, and the role of KRIT1 gene mutations could be hypothesized. Further studies are required.

## Conclusions

One of the cases illustrates an atypical presentation of epilepsy and cerebral hemorrhage with classical skin lesions in the context of blue nevus syndrome. As we can see, there are several clinical presentations in GI tract and CNS. The diagnosis remains challenging for unexperienced clinicians.

Genetic analysis is uncertain and further studies are needed. Most cases are sporadic, although some studies report autosomal dominant inheritance. Skin examination is of high diagnostic value and detailed neurological examination must be pursued. GI evaluation must be performed in all patients regardless of symptomatology.

Moreover, treatment of CNS lesions is scarce, and most clinicians support regular observation.

More information is warranted in order to elucidate the best medical and surgical approach for this potentially fatal syndrome.

## Data Availability Statement

The datasets generated for this study will not be made publicly available there no datasets.

## Author Contributions

AA: article correction and translation. G-RA: wrote the article, followed the patients, took the photos. P-AE: helped with the cases and writing. JA: made the main table, helped to translate. M-NI: made the dermatological diagnostic, Translating and writing. G-RA: images and table. SC: pathology processing and diagnosing. NA and VE: made the dermatological evaluation and follow up.

### Conflict of Interest

The authors declare that the research was conducted in the absence of any commercial or financial relationships that could be construed as a potential conflict of interest.
